# Miscellany

**Published:** 1911-12

**Authors:** 


### MISCELLANY

The U. S. Department of Commerce and Labor reports 5 aeroplanes exported to Canada in the first quarter of the fiscal year beginning July 1, 1911; and eight imported, seven from France and one from England, during the same period. The total value was over $50,000. A few years ago, air travel, except in a balloon at the mercy of the wind, was a joke. It is now an accomplished fact at a cost in money not much higher than for an automobile of medium grade and with every indication that the cost can be reduced below that of a run-about. The risk is scarcely higher though somewhat more prompt than for automobile racing. It is not unreasonable to expect that air travel will be on a practically useful and fairly safe basis, though with limits as to economic factors, within ten years. Speaking of risk, deaths due to automobile accidents have, for several years, been approximately equal to all those due to rail roads and trolleys, although probably hundreds of times greater per passenger mile. We are inclined to believe that the liberation of money by these deaths, has had a greater influence than anything else in postponing and mitigating the inevitable hard times due to rise of wages and curtailment on a large scale of the productiveness of labor.

---

The Perils of Bronchitis.—It is for the aged and anemic, that bronchitis has grave perils. The possibility of a severer infection grafting itself on the primary bronchial inflammation at once points to the wisdom of instituting treatment whose purpose will be to enrich the blood stream and add resistance to the tissues of the body.

Nutromel (Brown's Cotton Seed Oil Emulsion) possesses a peculiar fitness for this office; the prescriber may feel sure that the object of its administration will be fulfilled. Nutromel contains a large proportion of cotton seed oil, which is at last enjoying high favor as a reconstructive, and also the hypophosphites of lime, soda and manganese. It will aid materially in warding off the perils of bronchitis. A sample of Nutromel may be obtained by addressing Nottoc Laboratory, Atlanta, Ga.

---

Battle & Co. of St. Louis, have just issued No. 17 of their series of charts on dislocations. This series forms a most valuable and interesting addition to any physicians library. They will be sent free of charge on application, and back numbers will also be supplied. If you have missed any of these numbers, better write Battle & Co. for them before the supply is exhausted.

